# Looking back on 2 years of *Nursing Open*: looking forward

**DOI:** 10.1002/nop2.41

**Published:** 2015-11-30

**Authors:** Roger Watson

As *Nursing Open* enters its third year, it is worth recording that this January 2016 issue was published almost a month in advance. Little did we expect this kind of success when I was planning the launch of the journal with Wiley partner Griselda Campbell in a small tea room in a very wet and windy Cork, Ireland during the 2014 INANE conference. As ever, it is much easier to predict the past than to predict the future, but I think it is safe to say that we are now established and recognised as a reputable place to publish open access nursing articles. That is my prediction for the past. I hope and assume that the volume of submissions to *Nursing Open* will continue to increase in 2016 and beyond; and that is my prediction for the future.

As covered in several of my editorials for *Nursing Open*, the online open access publishing landscape is dangerous territory, especially for the unwary traveller. Predatory journals, hijacked websites and a plethora of poor quality publishing have not served to engender confidence. I am pleased that *Nursing Open* is proving to be a safe haven for those wishing to publish open access.

We continually strive to see how we can make the journal better, more prominent and achieve the various goals we have for *Nursing Open*. However, it strikes me that it is not an editorial team or a published that makes a journal better. Clearly, we have a part to play in setting standards and ensuring a good service to authors. However, it is authors who make journals better by choosing to publish their work in particular places. It is obvious that more authors are ‘voting with their manuscripts’ and choosing to publish in *Nursing Open*, and this is what increases our volume and visibility and, ultimately, levers up the quality of what we publish.

*Nursing Open* is promoted through the use of social media and authors and non‐authors can engage with us here. You can follow us on Twitter (@nursingopen), re‐tweet what you find interesting and also correspond with us there. We have a blog and there you can see my comment on some papers that we promote and it is also a place where you can contribute entries and comments relevant to the journal. I would also welcome occasional guest editorials.

Our first 2 years seem to have passed quickly; I look forward to the year ahead. If you need any information about *Nursing Open* then visit our website or email nursingopen@wiley.com.

